# Applying the Distributed Lag Non-Linear Model (DLNM) in Epidemiology: Temperature and Mortality in Mashhad

**Published:** 2019-11

**Authors:** Alireza ENTEZARI, Fatemeh MAYVANEH

**Affiliations:** Department of Geomorphology & Meteorology, Faculty of Geography and Environmental Sciences, Hakim Sabzevari University, Sabzevar, Iran

## Dear Editor-in-Chief

Effects of climate on human beings have been considered since Hippocrates who recognized climate as a factor in the prevalence of epidemics the mortality rate related to Cardiovascular Diseases (CVD) has been higher than Respiratory Diseases (RD). Sometimes, the effects of exposing a specific event do not only limited to the time of its occurrence and may show themselves with lag times. The Distributed Lag Non-Linear Model (DLNM) models the exposure-response relationship and consequently introduce a series of consequences caused by this exposure to events. In addition, determining the distribution of next effects after the occurrence of events (in lag times), this method is used as well. This method has been developed for time series data and is used in studies of design and structure of data, cohort, case-control or longitudinal studies. The present study used the climatological data of daily temperature (obtained from Synoptic Meteorological Station of Mashhad) and the rate of mortality caused by all diseases in Mashhad, Iran, (obtained from Organization of Municipality Paradises) in the period from 2004 to 2013.

**Fig. 1:**
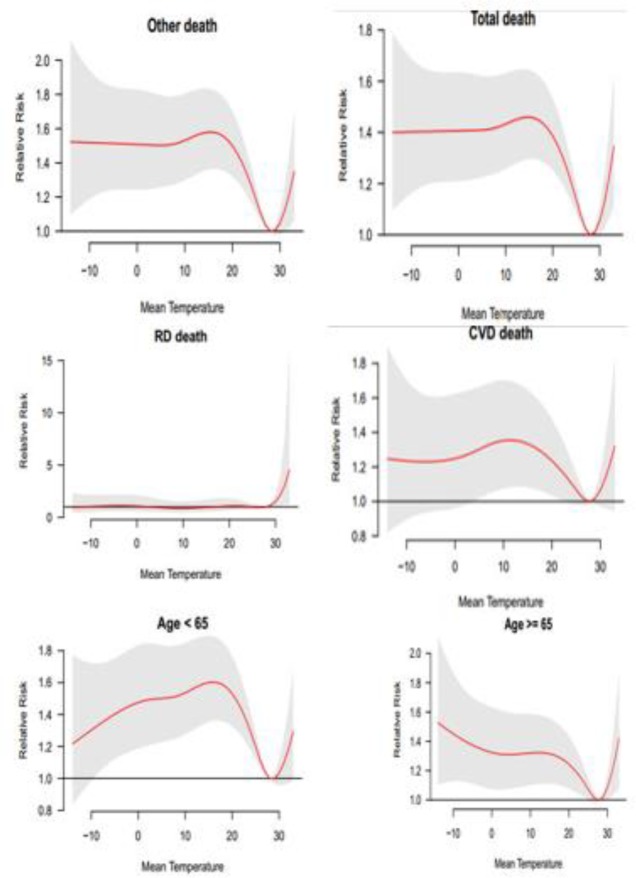
general estimation of effects of temperature on different types of mortality using Natural Cubic Spline– Natural Cubic Spline (DLNM)

The results obtained from investigations of the relationship between mortality and minimum and maximum temperature (Fig. 1) indicate the existence of a positive and significant correlation between total mortality rate in all groups and environmental factors. Temperatures higher than 30 °C increases the relative risk of mortality in both groups of diseases. The relative risk mortality in cardiovascular diseases is positively correlated with minimum temperatures. In the group aged below 65 yr, the relative risk of mortality in minimum temperatures increases up to 20 °C, and then the relative risk decreases up to 30 °C. After that, the mentioned temperature increases the relative risk of mortality from maximum and hot temperatures. In the group aged above 65 yr, the relative risk of mortality in minimum temperatures firstly increases and then gradually decreases up to 29 °C, and then in higher temperatures, the relative risk increases. In the below figure, the red line shows the relative risk and grey lines indicates the upper and lower bounds.

### Investigating the lagged temperature-mortality relationship

In investigating the exposure-response relationship, the issue of lags for obtaining acceptable results is considered.

**Fig. 2:**
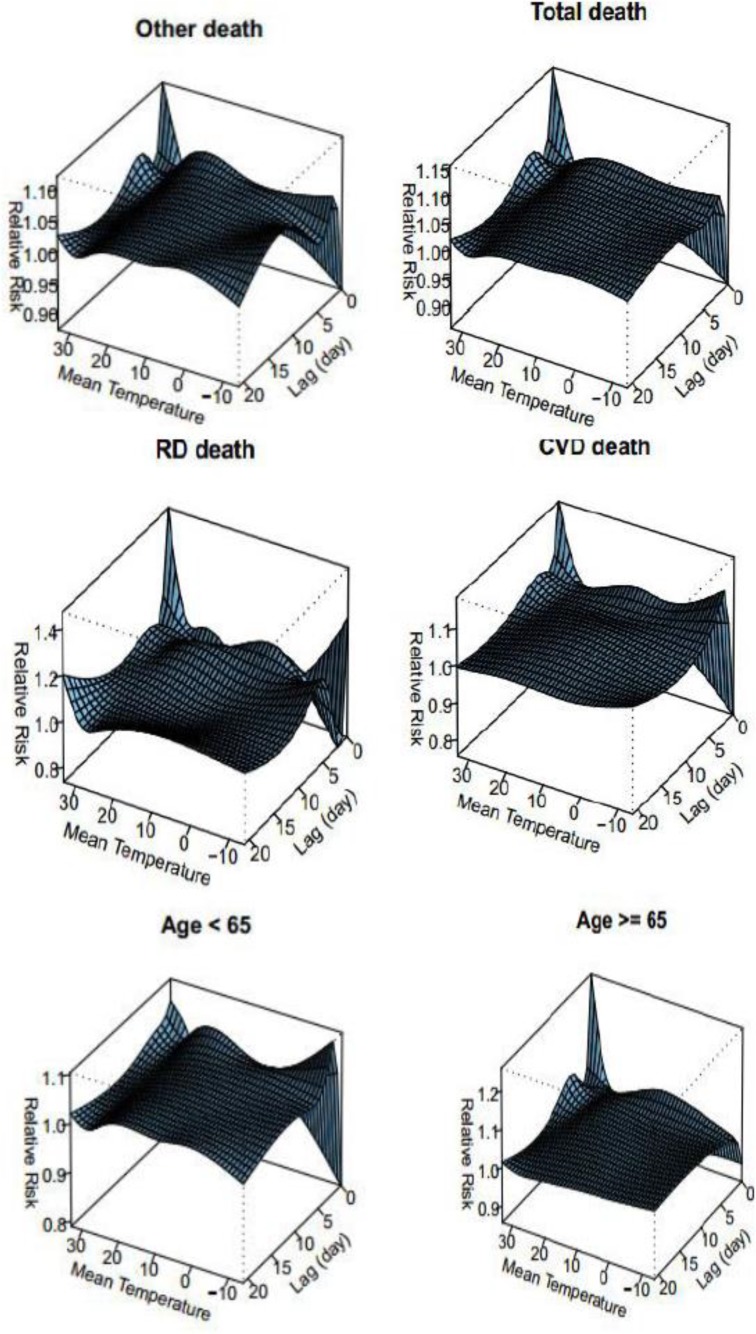
The relative risk (time lag) of different types of mortality using Natural Cubic Spline–Natural Cubic Spline (DLNM)

In the present study, to investigate real effects of temperature on mortality, the mortality lag time is used. As indicated in Fig. 2, in all mortality groups, the time lag from 3 to 5 d has the highest relative risk of mortality in temperatures from 20 to 30 °C and with the time lag from 5 to 10 d in temperatures from −5 to −10 °C. In the CVD group, the time lag with 2 or 3 d have been with the highest relative risk of mortality in minimum temperatures.

In addition, in the RD group, the time lag with 3 to 5 d have been with two maximum values of the relative risk; one in minimum temperatures higher than −10 °C and the other is between 20 to 30 °C. The existence of the time lag in the temperature-mortality relationship in the group aged below 65 yr indicates the maximum increase in the relative risk with the time lag from 0 to five days, and in case of the group aged above 65 yr, the time lag in the existence of the relationship can be neglected. Heat stresses have the highest relationship with mortality. The effects of cold weather on occurring RDs and CVDs and mortality caused by them (1–3). In addition, the fibrinogen levels have a reverse correlation with temperature so that with the increase in the fibrinogen levels, the degree of seasonal respiratory infections increases (4). The results of the present study indicated that there is a strong and positive correlation between temperatures higher than 30 °C in all types of mortality specially RDs and CVDs mortality. In addition, in temperatures higher than 30 °C, the relative risk of mortality in the two groups increases. The relative risk of mortality in CVDs has positive relationship with minimum temperatures.

In addition, in the group aged below 65 yr, the relative risk of mortality increased from minimum temperatures up to 20 °C, and then the degree of the relative risk reduces up to about 30 °C. After the mentioned temperature, the risk of mortality caused by maximum temperatures increases. In the case of time lags and the temperature-mortality relationship, the results indicate that in the CVDs group, the time lag with 2 or 3 d have been with the highest relative risk of mortality in minimum temperatures. In addition, in the RD group, the time lag with 3 to 5 d have been with two maximum values of the relative risk; one in minimum temperatures higher than −10 °C and the other is between 20 to 30 °C.
